# Pathogenesis of Noonan Syndrome is Modulated by NOC2L, a Novel Interactor of LZTR1 Leading to Impaired P53 Signalling

**DOI:** 10.1210/clinem/dgaf602

**Published:** 2025-11-01

**Authors:** Sumana Chatterjee, Miho Ishida, Débora R Bertola, Juliana Chizo Agwu, Carles Gaston-Massuet, Liam J McGuffin, Helen L Storr, Avinaash V Maharaj

**Affiliations:** Centre for Endocrinology, William Harvey Research Institute, QMUL, London, EC1M 6BQ, UK; Centre for Endocrinology, William Harvey Research Institute, QMUL, London, EC1M 6BQ, UK; Department of Paediatrics, University of São Paulo, São Paulo 05403-000, Brazil; Institute of Clinical Sciences, College of Medicine and Dental Sciences, University of Birmingham, Birmingham, B15 2TT, UK; Centre for Endocrinology, William Harvey Research Institute, QMUL, London, EC1M 6BQ, UK; School of Biological Sciences, University of Reading, Reading, RG6 6AS, UK; Centre for Endocrinology, William Harvey Research Institute, QMUL, London, EC1M 6BQ, UK; Centre for Endocrinology, William Harvey Research Institute, QMUL, London, EC1M 6BQ, UK

**Keywords:** Noonan syndrome, RAS/MAPK, LZTR1, NOC2L

## Abstract

**Introduction:**

Monoallelic dominant-negative *LZTR1* gene variants have been implicated as a cause of Noonan syndrome resulting from hyperactivation of canonical RAS-MAPK signalling. Missense *LZTR1* variants have been associated with defective ubiquitination theoretically leading to increased Ras substrate availability and altered p53 signalling. We investigated the role of LZTR1 in this pathway.

**Methods:**

Single nucleotide substitutions were generated by mutagenesis of an N-terminal MYC tagged-*LZTR1*-cDNA. Wild-type and variant constructs were expressed in mammalian cells and lysates prepared for phosphoproteomics. Analysis of transcriptomic data was conducted using Ingenuity-Pathway-Analysis. Significant phospho-peptides, protein-protein interactions, and pathways of interest were probed using immunoblotting, immunofluorescence, nanoluciferase assays and in silico modelling.

**Results:**

Two heterozygous *LZTR1* variants, affiliated with short stature, were shown in vitro to be thermodynamically stable and associated with elevated pan-Ras levels. Phosphoproteomics revealed upregulation of the histone acetyltransferase inhibitor, NOC2L, in both variants. This finding, consistent on immunoblotting and immunofluorescence, was associated with impaired p53 acetylation. Major effectors of the DNA damage response were preferentially activated in *LZTR1* variants. Despite an apparent activation of the DNA damage response and diminished p53 activity, levels of LC3 and phosphorylated-p70 S6 kinase were increased. *In silico* structure modelling and nanoluciferase assays suggested that LZTR1 interacts with NOC2L, an interaction disrupted in both *LZTR1* variants.

**Conclusion:**

NOC2L and p53 form a complex that dictates p53 activation. We demonstrate a previously unknown interaction between NOC2L and LZTR1 and hypothesize that LZTR1 acts as a binding factor modulating activity of this complex. Because NOC2L negatively regulates p53, its upregulation leads to p53-mediated transcription inhibition. LZTR1 attenuation resulting from genetic mutations associated with Noonan syndrome, potentiate NOC2L activity leading to reduced apoptosis and a compensatory increase in autophagy.

Noonan syndrome (NS) (OMIM 163950) is a dominantly inherited multisystem disorder with an estimated incidence of 1/1000 to 2500 live births (1, 2). It is defined by a characteristic phenotypic triad of short stature, distinctive facial features (short/webbed neck, low-set posteriorly rotated ears, ocular hypertelorism, ptosis, down-slanting palpebral fissures), and congenital heart defects (pulmonary valve stenosis and hypertrophic cardiomyopathy in 60% and 20% cases, respectively). Other associated phenotypic features include cryptorchidism in males, skeletal abnormalities (pectus deformities and scoliosis), developmental delay, and a predisposition to myeloproliferative disorders. Up to 80% of patients have postnatal proportionate short stature (2).

NS is a clinical diagnosis and establishing the diagnosis can be very difficult, especially in adulthood, given variable phenotypic expression. Variants in more than 20 genes have been associated with pathogenesis of NS (*PTPN11, SOS1, SOS2, KRAS, NRAS, RIT1, RRAS, RASA1, RASA2, MRAS, RAF1, BRAF, MAP2K1, MAP3K8, SPRY1, MYST4, A2ML1, SPRED2, CBL,* and *LZTR1*) and account for 75% to 80% cases (3, 4). All causative genes for NS, except *LZTR1*, encode components or regulators of the well-studied RAS MAPK signalling pathway.

*LZTR1*, located on chromosome 22q11.21, was initially described as a putative transcriptional regulator acting as an adaptor protein for CUL3 ubiquitin ligase complexes (5). In humans, *LZTR1* mutations were first associated with DiGeorge syndrome (22q11.2 deletion syndrome), whereas somatic variants with loss of heterozygosity at 22q11 were identified in glioblastoma multiforme, a malignant central nervous system tumor (6). Subsequently, several studies demonstrated an association between heterozygous missense *LZTR1* variants and NS. To date, more than 70 cases of NS with pathogenic *LZTR1* variants have been described (7-12) However, biallelic recessive variants have also been identified in several extensive kindreds (7, 11). The majority of heterozygous NS-causing variants are missense and map to the Kelch domains of the protein, whereas biallelic variants may be frameshift, nonsense, missense, or splice-site and are distributed across all functional domains.

Dominantly inherited *LZTR1* variants do not significantly impair protein stability and subcellular localization but do enhance stimulus-dependent RAS-MAPK signalling (13). Contrastingly, recessive variants are thermodynamically unstable and do not impact MAPK signalling (13). The distribution pattern of dominant and recessive NS-causing *LZTR1* mutations and their differential impact on MAPK signalling supports a dominant negative role for the former and a loss-of-function effect for the latter.

LZTR1 has been shown to enable ubiquitination of RAS proteins via its complex with cullin3; its loss negatively impacts RAS ubiquitination (14). Impaired ubiquitination thereby increases the pool of RAS proteins and augments MAPK pathway activity.

We investigated 2 naturally occurring heterozygous *LZTR1* variants identified in 2 patients with NS, both with characteristic facial features of NS, cardiac abnormalities, short stature, and features of GH insensitivity. Interestingly, our studies have identified a previously undescribed interaction between LZTR1 and NOC2L. NOC2 like nucleolar associated transcriptional repressor (NOC2L) was recently identified as a novel inhibitor of histone acetyltransferase, thereby inhibiting histone acetylation (15, 16). Furthermore, studies have also shown that NOC2L is recruited by P53 to inhibit histone acetylation in P53-targeted genes (17). NOC2L overexpression can therefore lead to inhibition of p53-mediated transcription, thereby impairing the process of apoptosis and cell-cycle arrest. The identification of an underlying genetic mutation in NS provides a definitive diagnosis and has important clinical and therapeutic implications for patients.

## Materials and Methods

### Ethical Approval

Informed written consents for publication of clinical details, including indirect identifiers, were obtained from human research participants and their guardians. Participants consented to dissemination of anonymized clinical data in an open-access journal and were not compensated for their involvement. The study was approved by the Health Research Authority, East of England-Cambridge East Research Ethics Committee (REC reference 17/EE/0178).

### Genetic Sequencing

Whole exome sequencing was conducted using the Agilent SureSelect all exon V4 capture and paired-end (2 × 100) sequencing on an Illumina HiSeq 2000 at Otogenetics (Norcross, GA, USA). Common variants were filtered out by excluding those with an allele frequency of ≥0.1% in the 1000 genomes, ExAC, and the NHLBI exomes. Variants predicted damaging by SIFT (http://sift.jcvi.org), PolyPhen-2 (http://genetics.bwh.harvard.edu/pph2/), or Mutation Taster (https://www.mutationtaster.org/) were explored further.

### Generation of *LZTR1* Variant Constructs

The wild-type (WT) *LZTR1* mammalian expression cDNA clone (pCDNA-MYC-Hist-LZTR1) was kindly donated by Dr. Antonio Lavarone at the Institute for Cancer Genetics, Columbia University Medical Center, New York, USA. Our variants of interest: *c.466A > G*, p.K156E and *c.742G > A*, p.G248R were generated by Site-Directed Mutagenesis (QuikChange II XL SDM Kit, Agilent Technologies) as per the manufacturer's instructions. All constructs were verified by Sanger sequencing (primer sequences available on request).

### Cell Lines and Transfection Protocols

Human embryonic kidney (HEK 293T, ATCC CRL-3216TM) cells were cultured in DMEM high glucose (Sigma D5648) supplemented with 10% fetal bovine serum and 1% penicillin/streptomycin at 37 °C in 5% CO_2_. Transfection of cells was achieved using Lipofectamine 3000 according to the manufacturer's instructions.

### Immunoblotting

Protein lysates were quantified using a Bradford protein assay (Bio-Rad), denatured by the addition of Laemmli SDS sample buffer 6X (Thermo Scientific) and boiled for 5 minutes at 98 °C. A total of 20 to 30 μg of protein was loaded into wells of 4% to 12% (Bis-Tris) 1.0-mm precast gels (Invitrogen) were used before electrophoretic separation using MOPS SDS running buffer. Semi-dry protein transfer to nitrocellulose membrane was achieved using a Trans-Blot SD Semi-Dry Transfer Cell (Bio-Rad) at 15 V for 1 hour. The membrane was blocked with 5% fat-free milk in Tris buffered saline (TBS)/0.1% Tween-20 for 1 hour at room temperature. Primary antibody was added at a concentration of 1:1000 and anti-β-actin (1:10 000) used as a housekeeping control. Primary antibody incubation in blocking buffer or 5% BSA in TBS/0.1% Tween-20 was performed overnight at 4 °C on gentle agitation. The membrane was then washed for 10 minutes (3X) with TBS-Tween-20. Secondary anti-mouse, anti-rabbit, or anti-goat antibodies were added at a concentration of 1:5000 to antibody dilution buffer and at 37 °C for 60 minutes. The membrane was washed 3 times (10 minutes each) with TBS-Tween-20 and visualized with the LI-COR Image Studio software.

### Phosphoproteomics

*LZTR1* WT, variant (p.K156E and p.G248R), and empty vector constructs were transiently transfected into HEK 293T cells using Lipofectamine 3000 according to the manufacturer's instructions. After 24 hours, cells were lysed in urea buffer and homogenized by sonication. The insoluble material was removed by centrifugation and protein in the cell extracts was quantified. A total of 250 µg of protein was reduced and digested with trypsin. Peptide solutions were desalted with Oasis cartridges and phosphopeptides were enriched using TiO2 as previously reported (18-20). Phosphopeptide pellets were resuspended in reconstitution buffer (20 fmol/µL enolase in 3% acetonitrile [ACN], 0.1% trifluroacetic acid [TFA]) and loaded onto an Orbitrap Q-Exactive Plus mass spectrometer (Thermo Fisher Scientific) as previously described (18-20). Differences in phosphorylation patterns between WT, *LZTR1* variant, and empty vector constructs were reported as fold over WT and statistical significance for those changes assessed using unpaired 2-tailed *t*-tests. Further analysis of transcriptomic data was conducted using Ingenuity Pathway Analysis (http://www.ingenuity.com).

### Immunofluorescence

Mammalian cells seeded on glass coverslips (24-well plates) were transfected with WT and *LZTR1* plasmid constructs. After 48 hours, cells were fixed with 4% paraformaldehyde for 15 minutes at room temperature. Cells were washed 3 times in PBS and permeabilized with 0.5% Triton X-100 in PBS for 10 minutes. After further PBS washes, coverslips were incubated in Blocking buffer (1X PBS/5% goat serum/0.3% Triton X-100) at room temperature for 60 minutes. Primary antibody (mouse anti-myc, rabbit anti-Pan-RAS, rabbit anti-NOC2L, mouse anti-p53, mouse anti-CHK1, rabbit anti-ATM, rabbit anti-LC3B) reconstituted in dilution Buffer (1X PBS/1% BSA/0.3% Triton X-100 buffer) was added to cells and left at 4 °C overnight with gentle agitation. Cells were washed and incubated in appropriate fluorescent secondary antibody at room temperature for 60 minutes (protected from light). Coverslips were stained with DAPI and mounted on microscope slides.

### Protein Structure Modelling

The latest version of the MultiFOLD server (21) (https://www.reading.ac.uk/bioinf/MultiFOLD/) was used to model the quaternary structures from the WT sequences of LZTR1 with NOC2L as well as each of the LZTR1 variants with NOC2L. Each of the modelled complexes was visualized using PyMOL and colored by a chain identifier. The NOC2L chains from all models were superposed using the align command to orientate the modelled complexes in the same frame of reference. The disordered N and C termini on NOC2L were identified and removed from the visualization for clarity. The locations of the mutated residues were identified and highlighted. Finally, the models were raytraced and exported as high-quality images.

### NanoBiT Complementation Assays

Binary protein interactions were assessed with NanoBiT complementation assays (as previously described) (22) using NOC2L WT and LZTR1 WT/variant plasmids N terminally fused with NanoBiT fragments (SmBiT and LgBiT). HEK 293T cells (1 × 10^5^ cells/well) were seeded in clear bottomed 96-well white plates, and plasmids were reverse-transfected using Lipofectamine 3000 according to the manufacturer's instructions. DNA concentrations were optimized and determined to be 200 ng per well; 100 ng SmBiT-NOC2L and 100 ng LgBiT-LZTR1. After 48 hours posttransfection, cell culture medium was replaced with 100 µL NanoBiT assay buffer (pH 7.4, HBSS 1X, HEPES 24 mM, NaHCO3 3.96 mM, CaCl2 1.3 mM, MgSO4 1 mM, BSA 0.1%) per well and left for 1 hour at 37 °C in 5% CO_2_. Subsequently, 6 baseline luminescence readings were recorded using the CLARIOstar Multimode Plate Reader (BMG Labtech) followed by addition of 25 µL/well of Furimazine (Nanolight Technology) prepared in a 1:20 dilution using assay buffer. Luminescence readings were resumed and continued for 1 hour.

### Resource Identification Initiative

Mouse Anti-Myc tag Monoclonal Antibody, Unconjugated, Clone 9E10 (Abcam Cat# ab32, RRID:AB_303599), Pan Ras Monoclonal Antibody (Ras10) (Thermo Fisher Scientific Cat# MA1-012, RRID:AB_2536664), Rabbit polyclonal NOC2L antibody (Proteintech Cat# 28509-1-AP, RRID:AB_2881160), Human p53 (acetyl K382) antibody (Abcam Cat# ab75754, RRID:AB_1310532), Rabbit monoclonal ATM antibody [Y170] (Abcam Cat# ab32420, RRID:AB_725574), Mouse monoclonal p53 (1C12) antibody (Cell Signaling Technology Cat# 2524, RRID:AB_331743), Mouse monoclonal Chk1 (2G1D5) antibody (Cell Signaling Technology Cat# 2360, RRID:AB_2080320), Rabbit Phospho-Rad50 (Ser635) Antibody (Cell Signaling Technology Cat# 14223, RRID:AB_2798430), Rabbit Phospho-ADD1/ADD2 (Ser726, Ser713) Polyclonal Antibody (Thermo Fisher Scientific Cat# PA5-40276, RRID:AB_2608892), Rabbit monoclonal anti-Rad51 antibody (Abcam Cat# ab133534, RRID:AB_2722613), Rabbit polyclonal Cathepsin D antibody (Abcam Cat# ab72915, RRID:AB_2040714), Rabbit polyclonal p70 S6 Kinase Antibody (Cell Signaling Technology Cat# 9202, RRID:AB_331676), Mouse monoclonal Phospho-p70 S6 Kinase (Thr389) (1A5) (Cell Signaling Technology Cat# 9206, RRID:AB_2285392), Rabbit monoclonal LC3B (D11) antibody (Cell Signaling Technology Cat# 3868, RRID:AB_2137707), Rabbit anti-GAPDH antibody (ab9485, RRID:AB_307275), Mouse anti-beta Actin monoclonal antibody (ab6276, RRID:AB_2223210), IRDye 800CW Goat anti-Mouse IgG (RRID:AB_10793856), IRDye 800CW Goat anti-Rabbit IgG (RRID:AB_10796098), IRDye 680RD Goat anti-Mouse IgG (RRID:AB_2651128), and IRDye 680RD Goat anti- Rabbit IgG (RRID:AB_2721181).

## Results

### Clinical Phenotypes of the 2 Individuals Harboring *LZTR1* Variants

Both patients 1 and 2 had classical facial features of NS (hypertelorism with down-slanting palpebral fissures, ptosis, low-set posteriorly rotated ears, and webbed neck) (Table 1) and cardiac pathology; pulmonary artery stenosis (patient 1) and pulmonary valve stenosis with an affiliated atrial septal defect (patient 2).

**Table 1.** Clinical details of patients with LZTR1 variants at diagnosis

Patient	*LZTR1* variant	Age at genetic diagnosis/sex/ethnicity	Height (SDS)	NS features	Birth-weight SDS	Height velocity cm/year	GH peak (mcg/L)	Type of GH stimulation test and age	IGF-1 ng/mL (SDS)	IGFBP 3 mg/L (SDS)	Segregation
1	c.466A > G; p.K156E	4.0 years, M, British	85 cm (-2.3)	Classical facies*^a^*, webbed neck, pulmonary artery stenosis, right undescended testis, left inguinal hernia, developmental delay	-1.6	3.6	10.6	Arginine, 3.5 years	19.8 (-2.3)	0.8 (-3.0)	Adopted
2*^b^*	c.742G > A; p.G248R	11.4 years, F, Brazilian	131.5 cm (−2.1)	Classical facies*^a^*, webbed neck, pectus deformity, pulmonary valve stenosis, atrial septal defect, prominent corneal nerves	-2.9	5.0	11.8	Clonidine, 5.6 years	35 (-2.2)	2.4 (-1.2)	Mother and maternal grandfather

Abbreviations: F, female; IGFBP3, IGF-binding protein 3; M, male; N/A, not applicable; N/D, not done; ND, no data; SDS, SD score.

^*a*^Classical facies, hypertelorism with down-slanting palpebral fissures, ptosis, and low-set posteriorly rotated ears.

^*b*^Variant previously reported; age and height SDS are at presentation.

Patient 1, a 4-year-old British-Caucasian male, presented with: short stature (height SD score -2.3), brachycephaly, bitemporal narrowing, minor degree of fifth finger clinodactyly, a single palmar crease on the left hand, tapering fingers, convex fingernails, and widely spaced nipples. Additional features included right unilateral cryptorchidism, left inguinal hernia, and developmental delay. The patient was adopted; hence, genetic mapping was not possible. Biochemically, the patient demonstrated an adequate response to GHRH-Arginine provocation testing with a peak GH of 10.56 mcg/L but concomitant IGF-I and IGFBP 3 deficiencies (-2.3 and -3.0 SD score, respectively) suggested a degree of GH insensitivity.

Patient 2, an 11.4-year-old Brazilian girl with short stature (height -2.1 SD score) and a history of congenital heart disease, presented in early childhood with classic features of NS and superimposed pectus excavatum, lacrimal duct obstruction, and prominent corneal nerves. GH provocation (Clonidine) elicited a peak of 11.8 mcg/L in tandem with persistently low IGF-1 (-2.2 SD score) levels. Interestingly, the patient's mother and maternal grandfather also had clinical features of NS and short stature (height -2.5 and -2.9 SD score, respectively). The patient was treated with recombinant human GH (rhGH) for several years with no response and annual height velocities fluctuating between 5.0 and 7.0 cm/year, resulting in a final adult height of -2.6 SDS (age 18 years).

### Evaluation of Pathogenicity of Variants Using in Silico Prediction Tools

Next-generation sequencing revealed single missense *LZTR1* variants harbored by each patient (patient 1—*c.466A > G*; p.K156E and patient 2—*c.742G > A*; p.G248R). Segregation analysis revealed maternal inheritance of the p.G248R variant, which was highly penetrant and present in the proband's affected mother and maternal grandfather. Both variants were rare; the p.K156E variant was novel and p.G248R had a minor allele frequency (MAF) of 0.000003718 in the Genome Aggregation Database (gnomAD) with 6 reported heterozygotes classified as likely pathogenic based on ClinVar entries related to underlying Rasopathy (Table 2) (8, 9, 12, 23, 24). The p.K156E variant was predicted benign by PolyPhen2 but deemed deleterious and disease causing by SIFT and Mutation Taster, respectively. The pathogenicity of this variant according to the American College of Medical Genetics and Genomics and the Association for Molecular Pathology's standards and guidelines for the interpretation of sequence variants was deemed to be likely pathogenic (PM2, PM1_Moderate, PP3, and PS3). The p.G248R variant was predicted deleterious across all 3 computational platforms and likely pathogenic according to concurrent ClinVar entries.

**Table 2.** In silico predictions of the LZTR1 variants

*LZTR1* variant	GnomAD frequency	SIFT	PolyPhen2	CADD	Mutation Taster
*c.466A > G*; p.K156E	Not reported	0 (deleterious)	0.301 (benign)	25.3	Disease causing, amino acid sequence changed, protein features might be affected
*^a^c.742G > A*; p.G248R	0.000003718	0.001 (deleterious)	1.0 (damaging)	28.9	Disease causing, amino acid sequence changed, protein features might be affected

For amino acid substitutions: Using SIFT a score of <0.05 is predicted deleterious; conversely, using PolyPhen2, scores closer to 1 are more likely to be damaging with variants within the range 0.85-1.0 more confidently predicted to be damaging. Mutation Taster provided automatic assignation of variants as disease causing or as a polymorphism.

^*a*^Variant previously reported.

### *LZTR1* Variants are Thermally Stable and Associated With Increased Pan-RAS Levels

**Figure 1.**
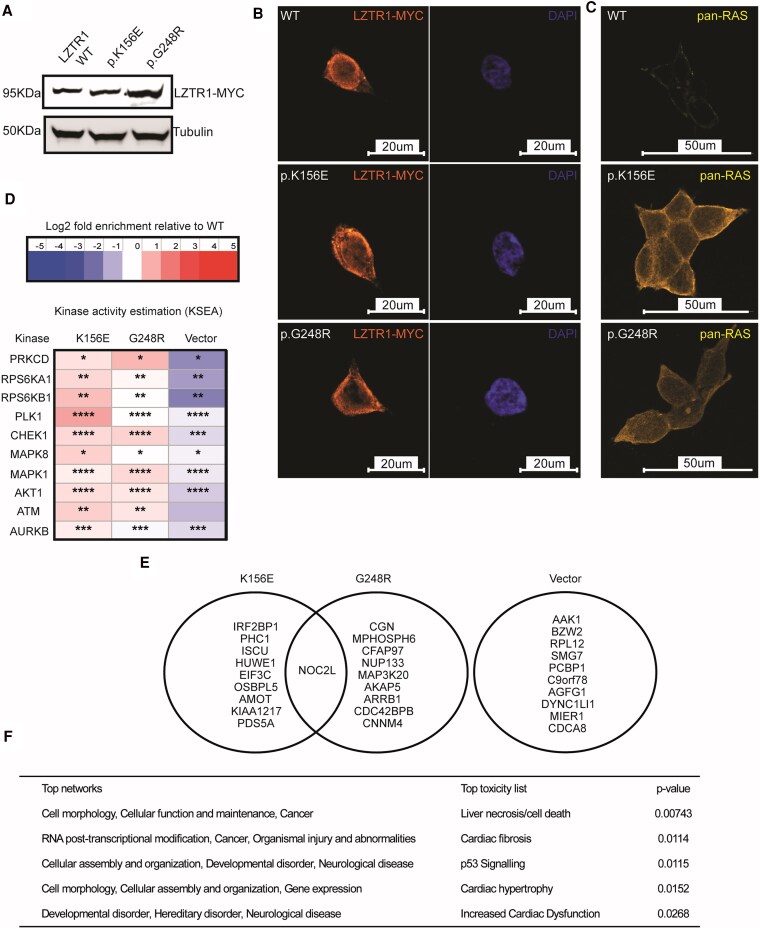
Characterization of *LZTR1* variants and phosphoproteomic analysis. (A) Immunoblotting showed stable expression levels of LZTR1 variants, p.K156E and p.G248R, analogous to wild-type LZTR1. LZTR1 expression localized to the nucleus and was broadly similar under all conditions (B). (C) Immunofluorescence highlighted increased pan-RAS levels for both variants detected on confocal microscopy. (D) Kinase activity estimation (KSEA) of 2 key variants, p.K156E and p.G248R, showed significant enrichment of DNA damage response (DDR)-related effectors, Ataxia Telangiectasia Mutated (ATM) kinase, and Checkpoint kinase 1 (CHK1). (E) Pathway analysis based on *P* value significance revealed a target histone acetyltransferase inhibitor, NOC2L (NOC2 Like Nucleolar Associated Transcriptional Repressor), upregulated in both variants. (F) Top networks and toxicity lists enriched in this dataset included cell morphology, cellular function, and maintenance/cancer as well as cell death and cardiac dysfunction. Functional pathway analysis was conducted using IPA (QIAGEN Inc., https://www.qiagenbioinformatics.com/products/ingenuity-pathway-analysis).

We assessed expression of both *LZTR1* variants following transient transfection into an *in vitro* HEK 293T reconstitution system. Both variants were well-expressed on immunoblotting, with levels demonstrably comparable to WT-LZTR1 (Fig. 1A). Furthermore, both variants localized to the nucleus similar to WT-LZTR1 (Fig. 1B); however, pan-Ras levels were markedly increased for both variants as evident on confocal microscopy (Fig. 1C).

### Phosphoproteomics

**Figure 2.**
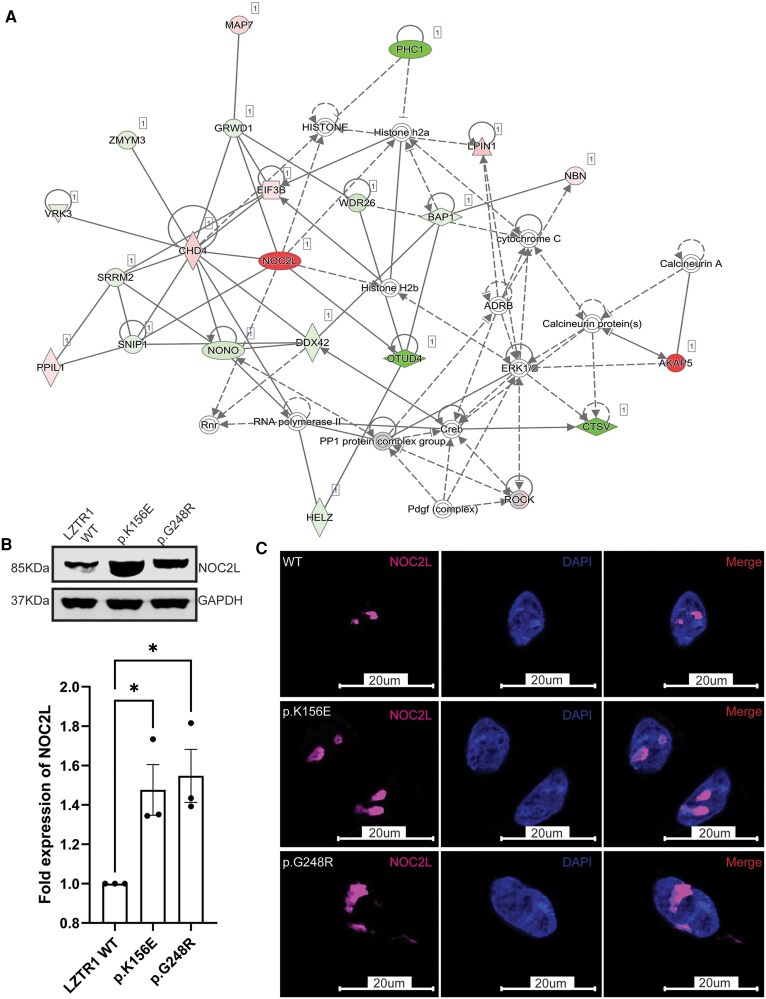
NOC2L is a novel target related to LZTR1 variation. (A) Protein interactome analysis of NOC2L and other differentially expressed targets using Ingenuity Pathway Analysis revealed multiple interactions with chromatin remodelling factors. (B, C) Increased levels of NOC2L were seen in both LZTR1 variants both by western blotting and immunofluorescence. Data are presented as the mean ± SE of the mean of 3 repeated measurements (3 independent replicates) (*P < .05).

We conducted phosphoproteomic analysis of LZTR1 mutants to identify unique transcriptomic signatures associated with LZTR1 variation. Kinase activity estimation demonstrated significant enrichment of Ataxia Telangiectasia Mutated (ATM) kinase and Checkpoint kinase 1 (CHK1), major effectors of the DNA damage response (DDR), which were both preferentially activated in LZTR1 variants (Fig. 1D). Ingenuity pathway analysis and filtering of molecules based on *P* value significance revealed a target histone acetyltransferase inhibitor, NOC2L (NOC2 Like Nucleolar Associated Transcriptional Repressor), upregulated in both variants (Fig. 1E). Top networks and toxicity lists enriched in this dataset included cell morphology, cellular function, and maintenance/cancer as well as cell death and cardiac dysfunction (Fig. 1F). Protein interactome analysis of NOC2L and other differentially expressed targets revealed a complex network involving Chromodomain-helicase-DNA-binding protein 4 and Histone H2B, nucleosome, and chromatin remodelling factors that suggest NOC2L may exert its effects via transcriptional regulation of several cofactors (Fig. 2A).

### NOC2L Localizes to the Nucleolus

Immunoblotting of lysates expressing WT-LZTR1 and variant constructs confirmed the phosphoproteomic findings, demonstrating robust detection of NOC2L in both variants when compared to wild-type (Fig. 2B). Immunofluorescence similarly highlighted an abundance of NOC2L for both variants, further denoting its nucleolar localization (Fig. 2C).

### NOC2L Upregulation Attenuates Acetylation and Expressivity of P53

**Figure 3.**
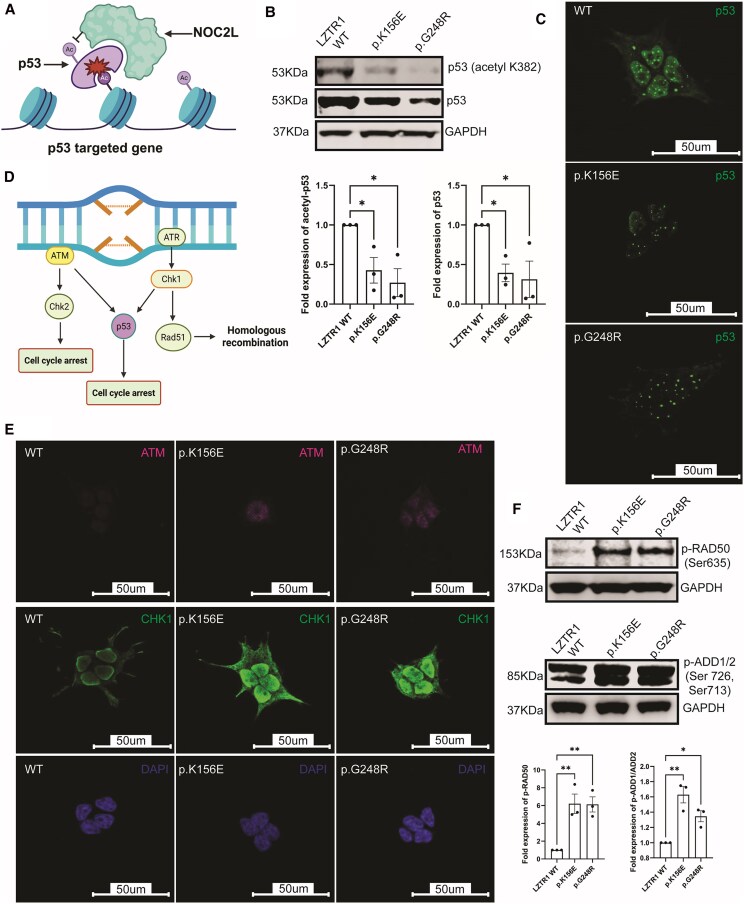
NOC2L upregulation dampens p53 activity. (A) In normal physiological states, NOC2L regulates the activity of p53 target genes and the acetylation and transcription of p53 itself. (B) For both LZTR1 variants, acetylation of the critical Lysine residue 382 of p53 and global levels of p53 were reduced when compared to WT-LZTR1. (C) Levels of p53 as detected on confocal microscopy were also reduced for both variants. (D). The DNA damage response involves several critical effectors such at ATM, CHK1, and Rad51 that induce p53-mediated apoptosis or DNA repair in response to chronic stress. (E) In LZTR1 variants, the kinases ATM and CHK1 were increased in both variants as probed by immunofluorescence. (F) Activity of both kinases ATM and CHK1 were probed by phosphorylation of 2 key substrates (Rad50, serine 635) and (Adducin1/2, serine 713/726), respectively. Levels of these phosphorylated substrates were increased for both LZTR1 variants. Figures 3A and 3D were created in BioRender (Maharaj A. (2025) https://BioRender.com/p19j619, Maharaj A. (2025 https://BioRender.com/j82t350). Data are presented as the mean ± SE of the mean of 3 repeated measurements (3 independent replicates) (*P < .05, **P < .01).

Previous work has intimated that physiological levels of NOC2L inhibit acetylation of p53-targeted gene promoters and inadvertently p53 itself (17) (Fig. 3A). Hence upregulation of NOC2L should theoretically demonstrate opposing functions. We probed acetylation of p53 lysine residue 382, critical for activation of p53 activity, via immunoblotting of WT-LZTR1 and variant expressing lysates. Levels of acetylated lysine 382 were reduced for both variants when compared to WT (Fig. 3B) and were concordant with reduced global levels of p53 as visualized by immunoblotting (Fig. 3B) and confocal microscopy (Fig. 3C).

### The DNA Damage Response is Activated Downstream of LZTR1 Variation

**Figure 4.**
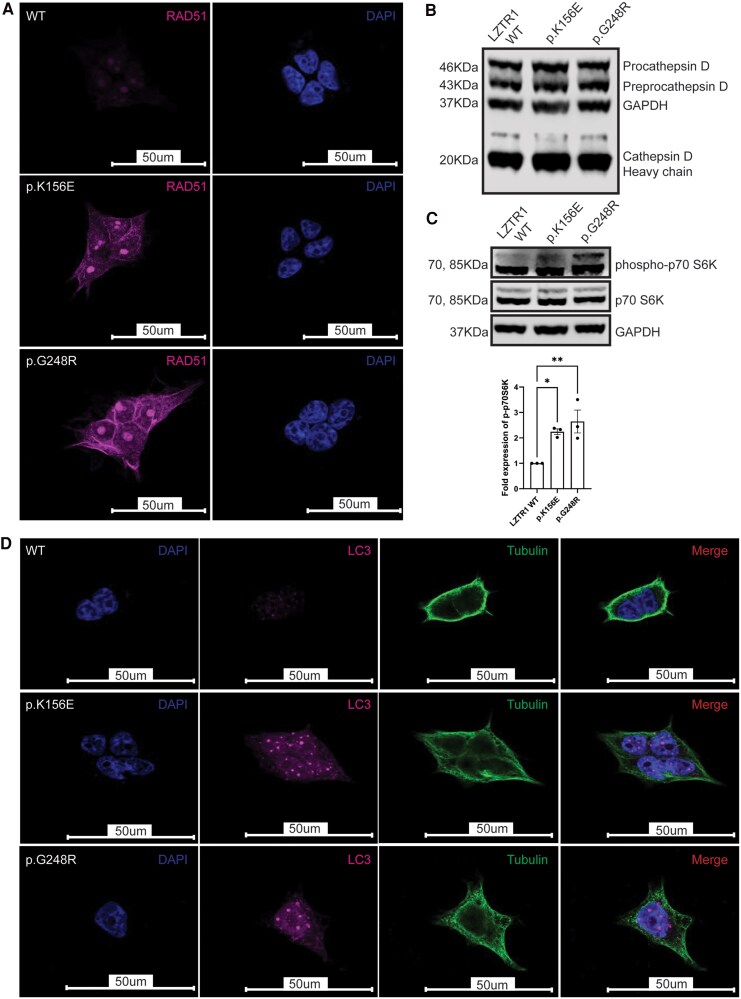
LZTR1 variants are associated with homeostatic disturbances between apoptosis and autophagy. (A) Expression levels of Rad51, a protein responsible for DNA repair, were increased for both LZTR1 variants. (B) Despite this relative increase in DNA damage markers, apoptotic signalling remained static as evidenced by equivocal levels of Cathepsin D for both WT-LZTR1 and mutant constructs. (C) Phosphorylated levels of downstream MTOR effector and marker of autophagy, p70 S6kinase, were increased for both LZTR1 variants. This correlated to a similar increase in LC3 levels detected upon immunofluorescence (D). Data are presented as the mean ± SE of the mean of 3 repeated measurements (3 independent replicates) (*P < .05, **P < .01).

The DDR involves several effectors such as ATM kinase and CHK1 that culminate in p53 activation or increased transcription of Rad51 to initiate homologous recombination (Fig. 3D). Global levels of ATM and CHK1 appeared enhanced in both LZTR1 variants (Fig. 3E), whereas 2 major substrates representative of their kinase activity, Rad50 and Adducin (ADD1/2), were preferentially phosphorylated in both variants, via residues Serine 635 and Serine 713/726, respectively (Fig. 3F). Furthermore, increased levels of Rad51 were noted on immunofluorescence of variant constructs relative to WT-LZTR1, when expressed in mammalian cells (Fig. 4A).

### Attenuated P53 Activity Leads to Compensatory Activation of Autophagy Signalling

The reduction in p53 expression was further associated with equivocal activity of apoptosis regulator, Cathepsin D in WT-LZTR1 and variant constructs as evidenced by commensurate expression of pro-and pre-procathepsin D levels (Fig. 4B). This apparent impasse in apoptotic activation despite a relative increase in the DDR was associated with increased markers of autophagy. Immunoblotting of WT-LZTR1 and variant constructs revealed increased phosphorylation of the threonine 389 residue of p70 S6kinase in variant constructs when compared to WT (Fig. 4C). Phosphorylation of this residue is critical for kinase activation and reflective of increased autophagic flux (Fig. 4C). This effect correlated to increased LC3 levels in LZTR1 variants detected upon confocal microscopy (Fig. 4D).

### LZTR1 Directly Interacts With NOC2L

**Figure 5.**
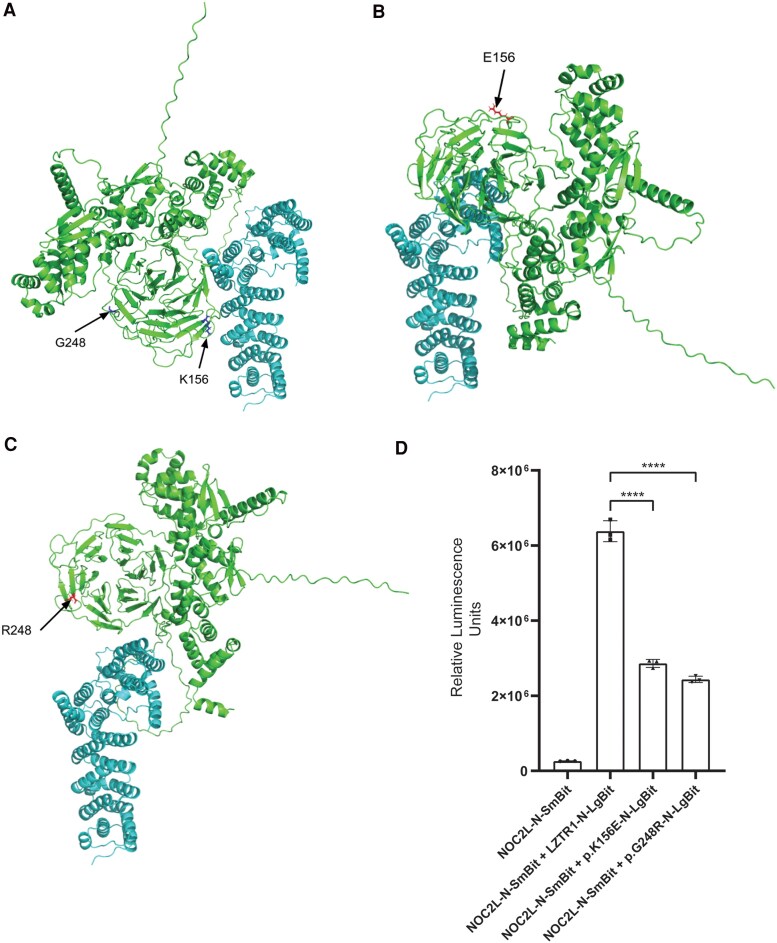
LZTR1 directly interacts with NOC2L. MultiFOLD2 models showing interactions between LZTR1 and NOC2L. NOC2L in cyan is kept in the same frame of reference and LZTR1 variants are shown in green. The disordered N and C termini on NOC2L have been removed for clarity. (A) Wild-type LZTR1 with NOC2L (plDDT = 0.90, pTM = 0.59). (B) LZTR1 mutant p.K156E with NOC2L (plDDT = 0.90, pTM = 0.59) (C) LZTR1 mutant p.G248R with NOC2L (plDDT = 0.89 pTM = 0.58). (D) NanoBit complementation assays demonstrated blunted interaction between NOC2L and LZTR1 variants p.K156E and p.G248R when compared to WT-LZTR1 (*P* < .0001). Ordinary 1-way ANOVA was used for statistical analysis with multiple testing corrections performed using Sidak's test. Data are presented as the mean ± SD of 3 repeated measurements (3 independent replicates).

*In silico* quaternary structure prediction using MultiFOLD2 demonstrated confidently modelled interactions between LZTR1 and NOC2L (Fig. 5A). The models showed different relative binding orientations for the LZTR1 mutants vs WT, suggesting alternative binding modes with NOC2L (Fig. 5B and 5C). Although the mutated residues in the models are not located within the binding interfaces, they may have downstream conformational effects. The model quality scores were marginally reduced for the p.G248R mutant, indicating a lower confidence in the interaction (Fig. 5B and 5C). To validate possible predicted protein-protein interaction between both key targets, Nanoluc Binary technology (NanoBit) was used. Complementation assays using Reporter-tagged target proteins demonstrated robust interaction between LZTR1 and NOC2L, as evidenced by detectable luminescence (Fig. 5D). Furthermore, this interaction with WT NOC2L was disrupted for both LZTR1 variants, with the greatest effect on p.G248R (Fig. 5D).

## Discussion

LZTR1, an adaptor protein for the CUL3 ubiquitin ligase complex, is implicated in NS yet its mechanism of action is not fully understood. *LZTR1* variants causing autosomal dominant NS likely inhibit binding to either RAS and/or other target substrates, thereby impairing their ubiquitination and degradation. Our missense variants (p.K1563E and p.G248R), are stably expressed and consistent with previously published work, associated with enhanced RAS activity (14). The homogeneous nature of dominant NS-causing *LZTR1* variants (ie, missense changes) and their clustering within the Kelch repeats strongly suggest a specific disruptive effect of variants on domain function and protein-protein interaction(s). Interestingly, we have identified NOC2L as a novel interactor of LZTR1 and whose levels appear to be enhanced in the setting of *LZTR1* variation.

Phosphoproteomic analysis of LZTR1 variants revealed specific phosphopeptide signatures indicative of upregulation of the DDR. This was consistent with previous data demonstrating activation of the DDR and chromosomal instability in association with germline *LZTR1* variants (25, 26). Key kinases involved in this pathway (ATM, CHK1) were upregulated with increased phosphorylation of respective target substrates, Rad50 and Adducin1/2 demonstrated by immunoblotting. A further marker of DNA damage and inducer of homologous recombination, Rad51 was shown to be increased in both LZTR1 variants when compared to WT. It is entirely possible that *LZTR1* variation may trigger genotoxic stress and an overall propensity to chronic DNA damage and increased risk of tumorigenesis (27).

We also identified NOC2L upregulation which was concordant in both *LZTR1* variants. NOC2 like nucleolar associated transcriptional repressor (NOC2L) is an endogenous inhibitor of histone acetylation and negatively regulates p53 activity (17). p53 directly interacts with NOC2L, facilitating its recruitment to inhibit transcriptional activation of p53-target genes; in the process, p53 is itself modulated by NOC2L, which inhibits its acetylation and subsequent activity (17). When compared to WT-LZTR1, both variants had attenuated acetylation of Lysine residue 382 and globally reduced levels of p53. The implication that apoptosis induction was impaired, despite activation of the DDR, was supported by relatively unaltered levels of apoptotic marker Cathepsin D across both LZTR1 mutants. This apparent blockade in apoptosis was compounded by an enhanced autophagic response in both LZTR1 variants, which demonstrated heightened LC3 levels and robust phosphorylation of threonine residue 389 of p70 S6Kinase, a downstream effector of mTOR whose activation correlates to an increase in autophagy. Previous work has demonstrated that transgenic expression of the human NS-associated *PTPN11* variant (*c.236A > G*, p.G79R) in murine endocardial cushions phenocopied the human cardiac valvulopathy resulting in increased cell proliferation and enlarged cushions due to reduced apoptosis (28). This suggests that multiorgan pathology in NS may be related to attenuated apoptotic signalling. Our findings highlight an increase in autophagy markers either as a compensatory response to diminished p53 activity or reflective of a general disruption in cellular homeostasis (29). Disequilibrium between both processes has been linked to a propensity to tumorigenesis, an established risk for patients with NS (30, 31).

Under normal physiological conditions, NOC2L is involved in embryogenesis, lymphopoiesis, and epidermal development likely mediated via its interaction with the closely related proteins, p53 and p63 (17). It is also implicated in carcinogenesis because of its diametrically opposing effect on p53 tumor suppressive activity (15, 17). Interestingly, Chinton et al reported 5 individuals with NS, harboring the *LZTR1* variant (*c.742G > A*; p.G248R) seen in patient 2, one of whom developed acute lymphoblastic leukemia (12). We report an upregulation of NOC2L in association with dominantly inherited missense *LZTR1* variants. Furthermore, we demonstrated a direct interaction between both proteins using in silico predictive tools and in vitro nanoluciferase technology; an interaction disrupted upon *LZTR1* variation. We hypothesize that WT LZTR1 may ubiquitinate NOC2L and regulate its activity, an effect lost due to *LZTR1* mutations leading to NOC2L hyperactivation. Given its involvement in cell-cycle regulation, NOC2L may be a potential regulator of organogenesis downstream of or in tandem with LZTR1, although further work is needed to characterize this effect.

There have been sporadic reports of affiliated GH deficiency (7, 10, 11), however, to date, GH insensitivity associated with *LZTR1* variants has not been reported and no underlying mechanism has been elucidated. Our patients both demonstrated biochemical evidence of IGF-I deficiency associated with sufficient GH levels and short stature (height SD score < -2). However, short stature in NS often exhibits incomplete penetrance (8, 9, 12, 23, 24). Therefore, growth dysregulation might be influenced by other factors including environmental factors, genetic modifiers, or novel interacting partners such as NOC2L that modulate affiliated signalling pathways. Therefore, further *in vitro* and *in vivo* data are required to delineate these associations and identify common transcriptomic links affiliated with NOC2L that may be implicated in multisystem pathology. Human GH is the only licensed treatment for growth failure associated with NS; however, as demonstrated in patient 2, therapy can often be suboptimal.

Therefore, growth dysregulation secondary to germline *LZTR1* variants, appears to be complex and multifaceted. Our data suggest that functionally disruptive *LZTR1* variants activate the DNA damage response while attenuating p53 function, likely through enhanced expression of NOC2L. Previous studies have shown that DNA damage can elicit increases in GH secretion which then further suppress p53 action and lead to accruement of further DNA damage response elements (32). Our *in vitro* model suggests an imbalance between apoptosis and autophagy, favoring an increase in the latter. Murine studies have shown that IGF-I-deficient mice demonstrate an upregulation in autophagy (33). Although further work is needed to corroborate these findings, the possibility of partial or “nonclassical” growth hormone insensitivity in our patients may be due to a direct NOC2L-mediated impact on p53 activity and homeostatic dysregulation of cell death pathways leading to compensatory increases in GH secretion despite IGF-I insufficiency. Furthermore, the therapeutic management of postnatal growth failure in patients with *LZTR1* variants is not straightforward. rhGH has been largely ineffective in our patients; this lack of response also suggests a degree of GH insensitivity. Additionally, given the postulated disruption of p53 signalling and associated potential increased neoplastic risk in this patient cohort, rhGH may be contraindicated. In other genetic forms of Noonan's syndrome such as PTPN11 mediated Noonan's, inhibition of ERK1/2 activity promotes IGF-I increases in vivo (34). Similarly, pending further studies, targeted inhibition of NOC2L and/or recombinant human IGF-I therapy may be a rational approach for patients with *LZTR1* variant-related short stature.

Given its overarching multisystem pathology, the health burden of NS is significant and associated with high morbidity (35). Hence, uncovering the mechanistic pathways involved in disease propagation is integral to evolving patient care. We propose a new disease model for LZTR1-mediated NS in which LZTR1 is postulated to form an interacting partner of NOC2L, modulating its ubiquitination and hence stability. Missense variation in *LZTR1* disrupts this interaction leading to upregulation of NOC2L activity and repression of p53 transcription. This triggers a cascade that culminates in an apoptosis blockade and compensatory autophagy induction that perpetuates a state of chronic DNA damage. Currently, no inhibitors of NOC2L exist, but this may be an apt therapeutic target that warrants exploration should future work uncover a broadly similar role for NOC2L in other genetic causes of NS.

## Data Availability

No new software or code were generated for the purposes of this manuscript. The phosphoproteomic data analyzed during the current study are not publicly available but are available from the corresponding author on reasonable request.

## Abbreviations

ATM: Ataxia Telangiectasia Mutated
CHK1: Checkpoint kinase 1
DDR: DNA damage response
HEK: human embryonic kidney
NOC2L: NOC2 like nucleolar associated transcriptional repressor
NS: Noonan syndrome
rhGH: recombinant human GH
TBS: Tris buffered saline
WT: wild-type
